# Revival of Bioengineered Proteins as Carriers for Nucleic Acids

**DOI:** 10.1021/acs.bioconjchem.4c00079

**Published:** 2024-04-15

**Authors:** David Scherer, Michael Burger, Jean-Christophe Leroux

**Affiliations:** Institute of Pharmaceutical Sciences, Department of Chemistry and Applied Biosciences, ETH Zurich, Zurich 8093, Switzerland

## Introduction

**Figure 1:**
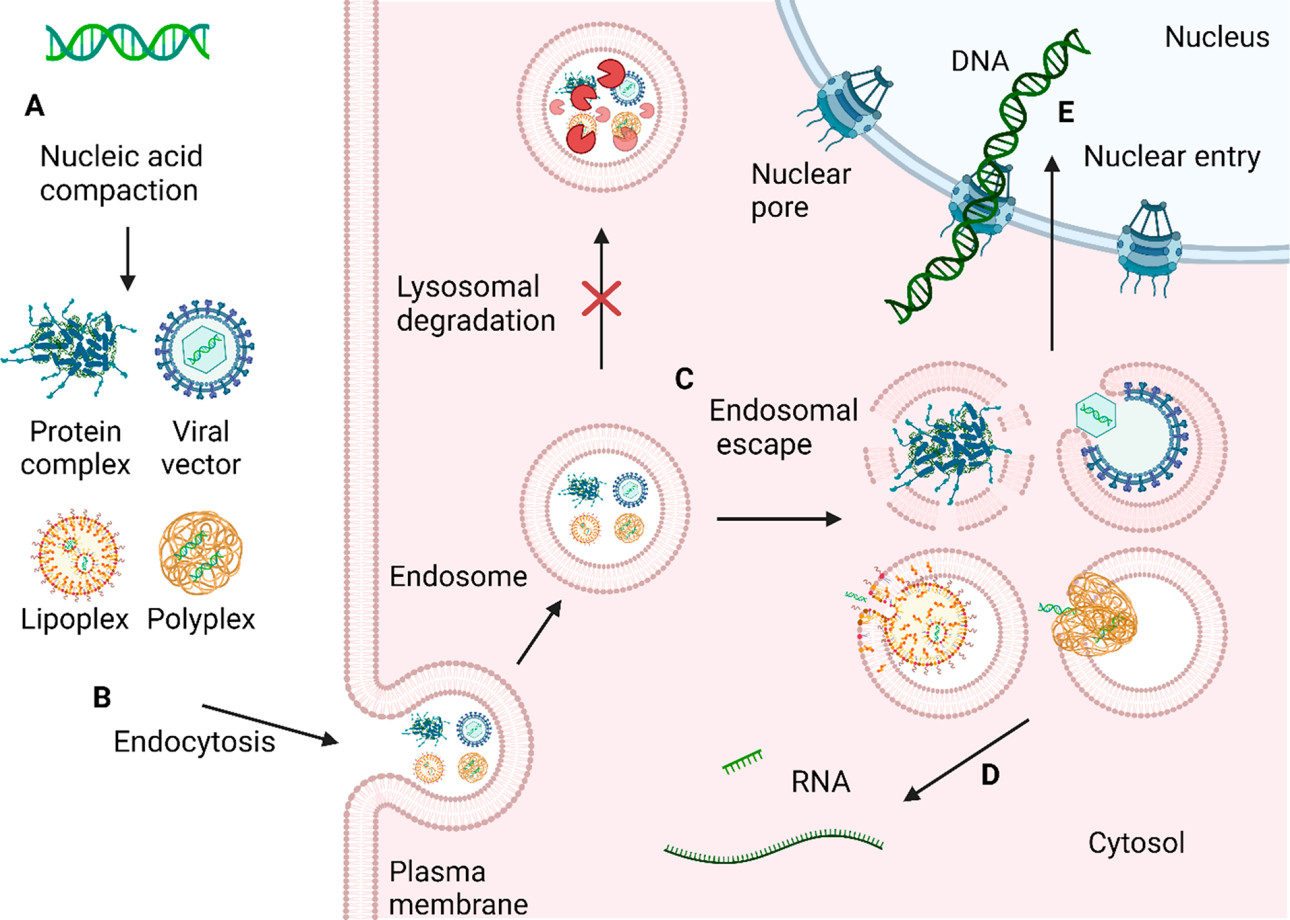
Nanocarriers compact the nucleic acids, shield them from body fluids, and permit their transport and uptake by target cells (A). Carrier endocytosis can occur spontaneously or be induced by a cellular receptor (B). Subsequently, the nucleic acid must escape the endosome to reach the cytoplasm (C, D). Finally, DNA needs to be protected and delivered to the nucleus (E). Created with BioRender.com.

Gene therapy can be defined as the manipulation of the cells of a patient by the introduction of genetic material or by making alterations to its genome, with the aim to treat a disease.^1^ Whether it is for the expression of a therapeutic protein or the correction of a defective gene, successful gene therapy relies on safe and efficient delivery systems.^2^ Compared to traditional drugs, nucleic acids are comparatively large molecules with a strong negative charge. Therefore, they are often delivered within nanosized carriers such as viruses, lipid- or polymer-DNA complexes (lipo/polyplexes).^3,4^ After injection, the carriers face the challenging environment of body fluids, including serum proteins such as antibodies and complement factors. This environment may destabilize the delivery system or prompt its rapid clearance. Subsequently, the carrier needs to reach and enter the target cells (Figure 1). Here, the plasma membrane is the most notable barrier.^5^ Current transfection agents generally address this hurdle via the process of endocytosis, followed by partial endosomal release and cytoplasmic entry of the cargo.^6,7^ Overcoming these innate barriers represents the primary challenge in the delivery of any nucleic acid. While RNA and oligonucleotides exert their activity in the cytoplasm, DNA-based therapeutics have, in addition, to access the nucleus. This is effectively countered by cytoplasmic DNA-defense systems such as the barrier-to-autointegration factor (BAF) and the cyclic GMP-AMP synthase (cGAS).^8−13^

To engineer an efficient gene delivery system, it is not sufficient to merely form nanoparticles that remain stable in serum, are taken up by cells, demonstrate endosomal escape, or protect the DNA cargo from the defense mechanisms in the cytoplasm. An optimal gene carrier must combine all those properties and possess adequate pharmacokinetic and biodistribution profiles.^14^ In a nutshell, the complex challenges in gene delivery require the development of a versatile molecular vector, and we believe that the extensive set of highly efficient proteins in nature stands among the most suitable building blocks of such a system. Here, we will briefly introduce the most commonly used gene delivery systems and argue for the advantages of proteins as the transfection agents of the future.

The early days of gene therapy were dominated by viral vectors, exploiting their inherent ability to transport genetic material into host cells. Viruses evolved to withstand body fluids and target specific cell types. They escape endosomes through precisely orchestrated mechanisms, which involve membrane-fusing protein complexes or phospholipases. These highly evolved functions make viruses the most effective vectors to date.^3^ However, viral systems suffer from persistent drawbacks including safety concerns, immunogenicity, inability to deliver chemically modified nucleic acids, and limited cargo capacity.^15−18^ Despite their drawbacks, significant breakthroughs were achieved such as the approval of the adeno-associated virus-based gene therapies Luxturna for the treatment of inherited retinal dystrophy and Zolgensma against spinal muscular atrophy.^19,20^ These advancements, however, were hard-won due to the high complexity and incomplete understanding of viral vectors that made progress challenging.^16,17,22^

The development of lipid nanoparticles (LNPs), a subtype of lipoplexes, as carriers for nucleic acid has been ongoing for several decades. Their relative simplicity in production allowed their rapid adoption as mRNA vaccines against SARS-CoV-2.^21^ The LNP lipid cores include permanent or ionizable cationic lipids that bind RNA in a charge-based manner.^22^ Additional functions are conveyed through incorporation of other lipid types such as PEGylated lipids to improve steric stability and circulation time. Despite their attractive properties, LNPs have limitations, which result in generally modest transfection efficacies. First, the process of endosomal escape of LNPs is still not well-understood and a major bottleneck in their performance.^23−25^ Inside the endosome, the lipids are thought to disturb and eventually rupture the endosomal membrane.^24,26−29^ The efficiency of the resulting cytoplasmic entry depends on the cargo size, lipid composition, and LNP nanostructure, but despite years of optimization, has remained between 0.3 and 3.5%.^25−28,30−35^ Difficulties addressing the endosomal escape issue suggest that lipids, at least by themselves, are not optimal to overcome this obstacle. Second, LNPs are usually developed with a strong focus on particle formation, cellular uptake, and endosomal escape. However, in the case of DNA delivery, the cargo should be further controlled inside the cytoplasm to mediate, for example, efficient nuclear uptake. This can barely be achieved with LNPs that get disrupted/degraded in the endolysosomal system. Third, the combination of LNPs with proteins that would enable additional functionalities is difficult, since organic solvents and harsh changes in pH are often required in their production. Lastly LNP formulations have been associated with side effects such as inflammatory responses and the production of anti-PEG antibodies.^36,37^

Polyplexes, most notably poly(ethylenimine) (PEI), have been extensively studied and display advantages and disadvantages similar to those of LNPs. PEI forms complexes with nucleic acids via electrostatic interactions. Subsequent to their cellular uptake it is hypothesized that the complexes escape the endosome due to the alleged proton-sponge effect and/or polymer mediated membrane destabilization.^38^ However, incompatibility with relevant serum concentrations and a direct relationship between endosomal escape rates and cytotoxicity limited the application of most polyplexes to *in vitro* transfections,^39^ where they remain nonviral vectors of choice due to their low cost.^40^ Recent efforts, however, managed to stabilize polyplex systems in higher serum concentrations (up to 50%) and reduce cytotoxic effects by, for example, the addition of PEG chains or carbohydrate moieties, resulting in improved *in vivo* delivery and higher translational potential.^41,42^

We believe that many of the drawbacks of the aforementioned systems might be addressed with transfection agents based on engineered proteins. The human genome alone contains the building plans for at least 10,000 proteins, each with highly evolved functions.^43^ These proteins can be modularly assembled, to build a molecular Swiss army knife, able to overcome multiple obstacles in the gene delivery process. In the 1990s researchers developed the first protein-based nucleic acid carriers.^44−46^ At the time, most efforts focused on DNA and how it could be compacted into nanoparticles with proteins. This was eventually achieved by using human genome organizing proteins, such as histones and histone fragments.^47^ Unfortunately, these early systems did not yet have the means to achieve efficient protein-based endosomal escape.^48^ Therefore, endosomolytic concentrations of Ca^2+^ and/or chloroquine were essential for transfection, largely preventing their use *in vivo*.^48−52^ On the other hand, *in vitro*, these systems fell short of the simplicity and affordability offered by poly- and lipoplexes. Following this, the discovery and characterization of numerous novel protein functions have armed researchers with potent building blocks for crafting protein-based carriers. Recent studies exploited the ability of proteins to be engineered both via rational design and directed evolution. Our laboratory reported a transfection system based on human mitochondrial transcription factor A (TFAM).^53^ TFAM possesses the ability to bind DNA with nanomolar affinity and organize it into nanoparticles.^54,55^ Point mutations were introduced to TFAM to enable the formation of the DNA/TFAM complexes (TFAMoplexes) in serum. To achieve endosomal escape of the TFAMoplex, a potent phospholipase from the bacterium *Listeria monocytogenes* (PLC) was fused to TFAM. The combination of the highly serum stable DNA complexation of TFAM and the efficacy of the phospholipase allowed superior cell transfection in 100% fetal bovine serum in comparison with Lipofectamine. This work illustrates the potential of exploiting highly evolved human proteins in biotechnological applications.

One of the most straightforward nucleic acid delivery methods is through antibody-oligonucleotide conjugates (AOCs). The simplicity of AOCs makes them readily adaptable, while the pool of available antibodies continuously expands.^56,57^ Since 2022, various AOCs entered phase 2 clinical trials for a variety of indications, such as myotonic dystrophy type 1.^58,59^ The principle of targeting carriers via antibodies and receptor-specific peptides is not limited to the delivery of oligonucleotides. Incorporation of these targeting moieties also showed increased cellular uptake and cell specificity for protein-based DNA and mRNA carriers.^60−62^ To enable cytoplasmic delivery multiple studies exploited the ability of pore-forming proteins to connect the endosomal lumen to the cytosol.^61,63,64^ For example, Wittrup et al., employed pore-forming perfringolysin O (PFO) as a potent endosomal escape agent in their protein-based siRNA carrier.^61^ The carrier complexed siRNA via the double-stranded RNA (dsRNA) binding domain of human protein kinase R. Efficient cellular uptake was achieved by fusion with engineered 10th type 3 fibronectin (Fn3). Target cell uptake was further enhanced by a cetuximab–Fn3 fusion protein previously shown to trigger internalization but not activation of epidermal growth factor receptors.^65^ Since the resulting system was vulnerable to serum, the dsRNA binding moiety was later exchanged for a viral dsRNA binding domain. Directed evolution in 55% mouse serum was performed on this domain to identify a dsRNA binder with enhanced serum tolerance.^66^ Finally, there has recently been growing interest in encapsulating nucleic acids within protein cages and virus-like particles (VLPs). Protein cages derived from human, bacterial, viral, and even denovo synthesized proteins were investigated for their ability to deliver nucleic acids.^67−72^ Researchers were able to show that nonenveloped VLPs resulting from the *in vitro* nucleic acid loading and reconstitution of viral capsids were able to transfect cells in cell cultures.^73,74^ While the encapsulation efficiency of genetic material in recombinant protein cages is quickly progressing, they currently lack mechanisms for efficient endosomal escape. Enveloped VLPs protect their nucleic acid cargo within a lipid bilayer and efficiently escape the endosome via incorporation of fusogenic membrane proteins.^75^ VLPs have been developed by David Liu et al. to codeliver effector proteins such as prime editors *in vivo*.^76^ However, the required assembly inside cells makes them more akin to viral vectors than other protein-based carriers.

## Challenges and Future Directions

While the prospects of bioengineered proteins in gene delivery are excellent, challenges, such as optimally addressing innate barriers and effective targeting, remain. Notably, the discovery of efficient endosomal escape agents, such as PFO and PLC, have shown promise. However, their origin from bacterial exotoxins introduces legitimate safety issues. Addressing immunogenicity concerns for viral vectors and LNPs has proven to be a complex endeavor.^18,36,77^ Similar immune responses to protein-based carriers need to be minimized. Such responses may occur, even with proteins of human origin, due to factors such as non-native folding, aggregation, and mislocalization of the protein. Considering that body fluids contain abundant endogenous proteins and protein complexes that do not provoke an immune response, it might be possible to engineer a protein complex to mimic the natural immune tolerance.^78^

Viruses protect and regulate their genome from the moment of formation until the delivery into the target organelle, e.g., the nucleus. Analogously, nonviral DNA delivery systems will have to achieve this as well in order to effectively cross the cytoplasm and shuttle the DNA into the nucleus. It is reasonable to assume that proteins can fulfill this task given their endogenous ability to transport large RNAs and other macromolecules through the nuclear pore. However, research on controlling transfected DNA within the cell is still in its early stages and will require substantially more research efforts to move forward.
